# Contemporary Knowledge on the Assessment of Temperament in Cattle and Its Impact on Production and Reproduction Including Some Immunological, Genetic and Metabolic Parameters

**DOI:** 10.3390/ani13121944

**Published:** 2023-06-09

**Authors:** Jędrzej M. Jaśkowski, Bartłomiej M. Jaśkowski, Magdalena Herudzińska, Oleksandra Tul, Marcin Ciorga

**Affiliations:** 1 Department of Diagnostics and Clinical Sciences, Institute of Veterinary Medicine, Faculty of Biological and Veterinary Sciences, Nicolaus Copernicus University, 87-100 Torun, Poland; 2 Department of Reproduction and Clinic of Farm Animals, Faculty of Veterinary Medicine, Wroclaw University of Environmental and Life Sciences, 50-366 Wroclaw, Poland; 3 Department of Basic and Preclinical Sciences, Institute of Veterinary Medicine, Faculty of Biological and Veterinary Sciences, Nicolaus Copernicus University, 87-100 Torun, Poland; 4 Department of Surgery and Obstetrics, Faculty of Veterinary Medicine, Poltava State Agrarian University, 36003 Poltava, Ukraine; 5 Department of Public Health Protection and Animal Welfare, Institute for Veterinary Medicine, Faculty of Biological and Veterinary Sciences, Nicolaus Copernicus University, 87-100 Torun, Poland

**Keywords:** behavior, temperament, cattle, reproduction, condition, production

## Abstract

**Simple Summary:**

The assessment of temperament has a long history in cattle. However, the tests used to evaluate it are often difficult and time-consuming and, therefore, rarely used in practice. Meanwhile, temperament affects many of the breeding parameters of cattle. The aim of the article is to present known and lesser-known methods of temperament assessment and to indicate its relationship and influence on production and reproductive parameters as well as on some immunological, genetic and metabolic parameters in cattle. The article presents the most commonly used methods of temperament assessment and discusses some of the issues surrounding them.

**Abstract:**

Temperament is associated with the well-being, health, production and reproduction of cattle. In order to increase the population of individuals with the desired temperament, its evaluation should be standardized and be made one of the obligatory elements of breeding and veterinary examination. A number of different tests are used for temperament assessment. In this article, the importance of temperament correlation with some metabolic, genetic, immunological, production and reproductive parameters have been shown, pointing at its influence on the economy and cattle handling. The most common methods for assessing the temperament of cattle are presented, including long-time scales of temperament assessment. At the same time, the relationship of the temperament of cattle with production efficiency, immunity and reproductive indicators has been shown, indicating that its correct assessment is an important aspect of the proper development of the herd and the associated economic growth.

## 1. Introduction

In the literature, consistent individual differences in behavior between individuals are referred to as temperament. This term concerns individual psycho-physical conditions on the basis of which mental actions are performed [1]. The oldest mentions of animal temperament date back to the late 19th century. In 1893, Waśniewski [2] defined it as follows, “temperament” is a living organism’s propensity to develop its reactions in a certain direction appropriate to it. The author distinguishes its three different types: choleric, sanguine and phlegmatic, attributing them to certain characteristic features of stature and behavior. However, such a division is used primarily in humans, and the temperament definition in animals during the last 100 years has undergone many changes. In the 1950s, laboratory animals that avoided human contact were described as “wild” and those that seemed unmoving/calm as “tamed” [3].

Several years later, temperament in cows was described by the degree of skittishness, excitability, apprehension or calmness [4]. However, most often, temperament was defined on the basis of the behavior of an animal reactivity in certain, specific situations [5]. Therefore, terms such as “handling temperament” and typical for cows’ “milking temperament” were coined [6]. At present, temperament in farm conditions is defined as a behavioral reaction observed during contact with humans [7,8,9]. Temperament can be defined also by animal’s reactivity to human contact and reaction to new objects or stressful situations [5].

One way in which temperament is commonly assessed in cattle is by evaluating their response to humans. A number of tests are based on the human–animal relationship. These tests are, for example, the reaction to a moving person, the reaction to a stationary person, and the reaction to handling. Avoidance distance in reaction to a human in a move is one of the more frequently used tests [10]. However, these methods are used primarily in beef cattle. The behavior of the animal could be repeatedly tested under strictly defined conditions, ensuring the repeatability of the assessment. Tests based on measurable features, e.g., Flight Distance (FD), have the greatest value. In dairy cattle, temperament is evaluated during the milking process and defined by the animal’s behavior and easy handling [11,12,13,14], while in beef breeds the most common method uses a chute and animal reaction after leaving it [15]. 

In the assessment of temperament, the descriptions of certain features of the animal personality are used. Temperament understood in such a way relates single animal characteristics to personality traits found in the entire population [16]. Tests used in case of cows are based on the subjective description of the animal personality. Records of these personalities used in described tests are not precisely defined, e.g., calm v. mild, docile, etc., which makes this evaluation system not entirely convincing and coherent. However, they are used as an element of temperament evaluation [17,18,19,20]. 

According to Buss [21], the term “temperament” is used to reflect genetic behavioral differences, while the term “personality” reflects non-genetic differences. Personality is defined as a correlated set of individual behavioral and physiological traits that are consistent over time and context [22]. Personality changes during the lifetime of cattle become consistent after puberty [17]. Due to lack of a clear boundary when it comes to cattle behavior, terms such as “personality” and “temperament” are sometimes used interchangeably [17]. Furthermore, similar tests are used to assess temperament and personality. For example, one of the most frequently used tests to assess personality in cattle is similar to the one used for assessment of temperament, i.e., exit time [18]. Moreover, in this article, the term “temperament” and “personality” are treated as identical, and hence in the following parts of the article, only the term temperament is used. It is worth emphasizing that the terms used to evaluate animal behavior are derived directly from analogous terms used in human psychology. A detailed description of these terms was presented, among others, by Finkemeier et al. [22]. However, in thecited paper, the authors focus mainly on definitions and concept of temperament without focusing on production and parameters that we included in our work.

In this review, we will discuss some of the common methods for assessing temperament in cattle and their influence on production and reproduction along with immunological, genetic and metabolic parameters. 

## 2. Temperament Assessment in Cattle

A number of cattle temperament assessment methods with different evaluation criteria have been developed. In most cases, they use some kind of scoring system. Among the most commonly used criteria there is chute exit velocity where the subject of examination is how fast the animal leaves the chute and if it demonstrates behaviors such as jumping, ambling or running [23,24]. Another widely used test is the chute score where the behavior of animal is rated while it stays inside of the chute [25]. Evaluation of temperament during milking [26,27] and weighing of the animal [28,29] are also considered useful tools by many authors. Many papers include the mixing of two or more assessment methods and evaluation criteria. [30,31]. There are also complex tests which are a sum of a seven-point evaluation of the vigor with which animal was moving and the rate of intensity of audible breathing. Additional points were awarded to animals that demonstrated kicking, bell ringing or kneeling, 0 points if no such behaviors were observed and 2 points if a given cow laid down. The sum of those points, according to the authors, seemed to be the best means of a comprehensive evaluation of an animals’ reaction to stress [32,33]. The most common tests and their evaluation rules are presented in Table 1.

One of the methods with high repeatability is the Flight Distance (FD) test, which enables the precise identification of excitable specimens [32]. The FD is the distance at which a cow retreats from an observer. If the animal did not withdraw when approached, the Flight Distance was 0 m. In one study, the average FD was 1.6 m (ranging from 0–6.5 m) [35,36]. However, the FD test is relatively difficult and time-consuming. For that reason, it is not easy to include it in routine management procedures. A more interesting solution is the Flight Speed (FS) test, also known as the Exit Velocity (EV) test [37,38]. It resulted from observing that some animals remained calm after stepping off the chute, while others left it much faster. For measuring purposes, a simple electronic system was used to allow the assessment of the time necessary to cover a given distance. The system consists of two beams of infrared light situated at a certain distance from each other that focused on reflectors with automatic turn-on and turn-off mechanisms after the beam is crossed by a passing animal. The time of covering a set distance in this way was registered in tenths of a second. In the beginning, the distance was about two meters but was later standardized and set at 1.7, 1.83 or 2 m [30,38,39]. Other temperament assessment methods include Parlor Leaving Speed (PLS) or Temperament Score (TS). The PLS is calculated as a specific distance covered by an animal divided by the time taken to cover it. Agravat et al. [40] proved that aggressive Gyr cows had significantly higher mean PLS as compared to docile and restless cows. The TS is given to the animals’ reaction after leaving the chute and entering one pen of the corral. The scoring system is based on a five-point scale where one point is given to the animal that walks slowly, while the highest score is given to the animal that runs non-stop during assessment and tries to attack the observer [41].

Analyzing the above-mentioned methods of assessing the temperament of cattle, it is clearly visible that there is no one universal way to assess it. The methods used differ in dairy and beef cattle, the amount of time needed to perform them is different, and not all of them can be performed in strictly defined handling conditions. Taking into account the significant influence of temperament on production, reproduction and health of cows, further research is needed to establish a simple, cheap and repeatable test feasible in all conditions of living cattle.

## 3. Physiological Markers Correlating with Temperament

Some of a stress markers also correlate with a temperament. Measurement of a stress marker, cortisol, in saliva, feces and hair using non-invasive methods has been described [42]. Excitable beef cows have higher plasma or serum cortisol concentration (CC) than calm cows [43]. Simultaneously, EV positive correlated with blood CC in a long-term examination of yearlings Brahman bulls on the initial and final (120 d) day of the experiment [38]. For this reason, its measurement was considered a valuable biomarker of stress and cow temperament. The weak point related to the determination of CC in the blood is the procedure of blood collection. It can cause a neuroendocrine response, which significantly affects the measurement result. It seems to be a better idea to measure cortisol in the coat. However, there were no differences in hair CC between calm and excitable cows [44]. The CC in the coat is considered an indicator of long-term stress [14]. 

Furthermore, measuring the heart rate and heart rate variability (HRV) is considered a non-invasive method helpful in temperament evaluation in beef and dairy cows [45,46,47]. Heart rate variability is a good indicator of stress and balance of the autonomic nervous system, and low parasympathetic activity is connected with higher emotional reactivity, and it is defined as the animal’s tendency to show more or less pronounced reactions to different fear-inducing situations [47]. However, no differences were noted in the heart rate or HRV parameters between groups with different temperaments during milking [46]. On the other hand, the heart rate in calm calves was lower than in other ones. Similarly, the body temperature which also correlated with temperament was lower in the calm group than in the nervous group [48,49]. 

To assess the emotional stress of cows, attempts were also made to use the measurement of the area of the visible white of the eye, nose and ear posture [50,51]. Sandem et al. [52] and Core eta al. [53] discovered that the percentage of white in the eye was higher than normal in cows that were frightened and lower when they had access to food. Measurement of visible eye white was highly repetitive and according to authors could be used as quantitative tool to asses temperament in dairy and beef cattle. 

In the other study, Lanier et al. [54] established that the degree of reaction to sudden movements and sounds might be positively correlated with temperament evaluation. 

However, these methods are of little practical importance and are not widely used. They are mostly treated as a supplement to scientific experiments. Therefore, further studies on their correlation with temperament are required. In the future, they may find wider use and practical importance.

## 4. Temperament—The Impact of Individual Characteristics, Living Conditions and Contact with Humans

Cattle temperament is hereditary to a moderate extent [39,55]. It was determined several years ago that the most serious problems with aggressive temperament in cattle in Europe were found in continental breeds of cattle because, as Grandin speculated, they were not reared in extensive pasture conditions where they had little contact with people. When animals were kept in intensive conditions, their temperament was masked because it was easier to tame them. Conversely, handling of cattle breeds that for centuries had been kept in half-extensive, primitive conditions was difficult and dangerous. Thus, they were often culled because of their excitable temperament [56]. In more recent studies, attempts to identify candidate genes that may affect the behavior of cattle have been made. Shen et al. [57] identified genes that could have a significant influence on animal behavior from 13 million single nucleotide polymorphisms (SNPs) from the whole genome of cattle. Based on the tissue expression profiles, the expression of some genes in the brain has been proved. One of them (SORCS3) is a postsynaptic modulator of synaptic depression and fear extinction and may play an important role in emotional regulation in cattle. Furthermore, the gene associated with the most traits in the novel subject test (SESTD1) was highly expressed in domestic cattle brains. Additionally, it was shown that at least seven SNPs connected with temperament determined in the studies by Jakimowicz et al. [58] were localized on the X chromosome. Moreover, in Brahman cattle, genomic regions and genes related to temperament have recently been identified [59].

Temperament can differ depending on age, sex, herd management and breed-dependent factors [7,55,60]. It was also pointed out that females with an excitable temperament showed a more masculine appearance [56]. They also vocalized more often [56]. Temperament scores differed significantly between male and female calves with females being more nervous [60,61]. The sex of cattle influences temperament even in the weaning period [62,63], and the correlations is also observed in the 18th month of life [37,60]. The differences in temperament may influence animals’ reactions to stress and fear factors [8,43,45]. Historical data from Australian research indicate that the temperament of Brahman cross-breeds is calmer than Shorthorns [33]. Moreover, cows that were heavier and horned had more nervous temperament than lighter and hornless [33]. A similar observation was made by Americans who attributed more nervous temperament and excitability to Brahman cross-breeds (≥25%) than to other cross-breeds that were free of Brahman genetics [62]. However, those studies have a territorial range limited to western and eastern part of Germany and cannot be considered descriptive for the entire population. Charolaise and Limousine cattle were more nervous than the cattle of Hereford. Moreover, German Angus and Hereford calves had the lowest scores in temperament evaluation, indicating that these breeds have a more favorable temperament [60]. On the other hand, no influence of cross-breeding between African breeds and between African and Brahman breeds on temperament has been observed [35,63].

Temperament has been linked to coat color in several studies. The first mentions of the coat color and temperament were presented by Keller in 1947 [64]. Generally, brown cattle were calmer than white or black. It is supported by newer research in which temperament evaluation based on EV proved that red Angus bulls were calmer than black bulls [63]. 

In the research based on temperament analysis during milking, on a five-point scale, only 24.07% of primiparous females were given the lowest score (determined as docile). In following lactations (second, third and fourth), docile animals were much more frequently observed (73.33, 57.40 and 59.37%, respectively) compared to other temperaments [65]. 

Animals’ temperament may also be related to the building they are handled in. Appropriately designed buildings make animal handling easier and at the same time influence cattle behavior. Nervous cows are more likely to be self-injured than calm ones [56]. 

Calmness is a desirable feature of livestock. It can be stimulated by appropriate handling that does not evoke fear. An effective, long-term breeders’ policy towards specimens with nervous temperament is (especially in the case of cattle) an appropriate selection and culling of animals on whom calming attempts are ineffective [1]. Constant contact with humans also has a certain influence on the disposition of cows. The cows which had frequent contact with people had calmer temperament [66]. Similarly, the excitable nature of cattle can be effectively obscured by calming [56]. Although it is proven that heifers have a certain ability to acclimatize to being handled by humans, and this manifested in the form of improved fertility [67], but this phenomenon was not observed among mature cows [68,69,70]. However, the Aberdeen Angus cows that had frequent contact with people had a calmer temperament. Moreover, the young animals were calmer and got more easily accustomed to working in chutes when they were reared together with cows of different ages [66]. 

## 5. Temperament vs. Productive Traits

Temperament in cows has an influence on many production aspects, such as immunity, carcass characteristics and ability to learn [19,30,71,72,73]. There was a relationship between temperament and fecal microbiota as well as serum and fecal metabolites of calm and nervous six-month-old Brahman bulls and heifers [74]. Excitable cattle are more prone to stress. Acute stress may influence gastrointestinal tract microbiota, leading to dysbiosis and production of metabolites, such as amines or indoles, which may be toxic and repress growth [75]. Some data show that cattle that were quieter and calmer during handling had greater average daily weight gains than cattle that became agitated during routine handling [61]. Calmer steers with slow EV gained 0.12 kg/d more than fast EV steers [76]. Other opinions on beef cattle are that it is difficult to determine a clear cause–effect relationship between those variables, such as body weight gain, temperament, stress and puberty attainment, but a relationship among these parameters is evident in which animals with more excitable temperament are, overall, counterproductive for the beef-producing chain [44]. 

Greater susceptibility to stress noted in the case of excitable cattle had an influence on milk and meat production as well as inhibited calf growth [55,61,77,78]. Cows’ temperament was also associated with metabolism, which depended on parity and temperament. Calmer cows had a higher milk production than their nervous counterparts [19,79,80]. However, there are also conflicting reports of a correlation between temperament and milk yield with lower production for calmer cows [81,82,83]. These papers showed that multiparous reactive cows had a greater milking frequency, milk yield, fat protein and lactose [81,82]. However, cows that were more fearful of humans (reactive toward the novel human) had reduced rest time compared with cows that scored low on this trait. On the other hand, cows that were calmer (during restraint) and investigative (toward the novel object) had higher grazing time, which likely contributed to their higher milk production compared with cows that scored low on this trait [82]. Cows that were more reactive to milking produced less milk than calm cows [19]. Although most papers show that dairy cattle in the milking parlor rated as nervous produced less milk, milked slower and had lower lifetime productivity [6,19,79,80,84,85], there are also reports saying that temperament does not influence cow milk production [86]. In the case of cattle, a selection that includes temperament while milking may be more effective than a selection oriented towards dominance [87]. 

There is a certain relationship between temperament and the number of somatic cells in milk of Jersey and Holstein Friesian breeds. Calm cows had a lower somatic cell count in comparison to those that were more nervous [88,89]. According to Van Doormaal [90], the speed of milking and excitable temperament may increase the rate of culling animals by as much as 2%. In addition, excitable cows cause difficulties with herd management and pose a greater threat to the farm personnel. According to Polish research, the most appropriate temperament is demonstrated by cows from Polish Red and Fleckvieh breeds, while the greatest nervousness while milking is shown by the Holstein Friesian breeds of both colors [91]. Cows with the desired temperament constitute about 84.7–88% of the total population, but the percentage of calm cows has been growing continuously over recent years from 2.98 to 6.85% [26]. The best lifetime efficiency and survivability were achieved by cows with a calm temperament. However, cows with moderate temperament and high-milking speed had higher daily efficiency, fat and protein content in milk in comparison to those with low-milking speed. At the same time, cows characterized by high-milking speed were also noted for their highest culling rate due to metabolic disorders and infertility [26]. Selection for calmer cattle could reduce physical activity and increase feed consumption, which may consequently improve feed conversion and promote growth, respectively [92].

## 6. Temperament vs. Carcasses and Meat Quality

Cattle with an excitable temperament had carcasses of lower quality as well as tougher, less tender meat than cattle with a calm temperament [61,62,93,94]. Evaluation of the emotional reactivity of cows may also serve as an indicator of stress reactions of cattle subjected to routine procedures in a slaughterhouse [95]. Simultaneously, cattle with more excitable temperaments are thought to have a stronger negative reaction to handling and suffer more adverse effects with subsequent negative effects on their performance, carcass and meat quality traits [30,96]. A good indicator of temperament and its influence on bullocks’ growth rate and tenderness of meat may be the concentration of lactic acid in peripheral blood [7,93]. Cattle classified as excitable presented greater amounts of carcass bruises [97]. Holmes et al. [98] reported that, during the exercise, heifers with excitable temperament had a greater increase in blood lactate (BL) concentration compared to heifers of calmer dispositions. More recent studies confirmed temperament’s impact on the BL, growth rate and tenderness of meat in Siamese sires of Simmental × Angus cattle. Steaks from steers in the medium BL classification were more tender than steaks from steers from the high BL classification. In other studies, Moura et al. [99] stated that serum CC and glucose levels correlate positively with beef cattle temperament and negatively with meat quality. At bleeding, an increase in glucose and CC was observed for storing periods longer than 24 h and 12 h, respectively. Storing for 48 h reduces meat tenderness and meat water-holding capacity [99]. However, results from the literature are inconsistent. In experimental conditions, restless and nervous animals had shorter carcasses and lower fasting weights than calm animals. Nevertheless, carcass yield in nervous animals did not differ from that of calm ones but was higher in comparison to restless animals [100]. Sant’Anna et al. [101] stated that excitable temperament in Nellore cows might have negative effects on some of the carcass and meat quality attributes assessed, mainly those related to muscle deposition on carcass and color gradients. The authors suggested that temperament assessment before the cattle entered the feedlot was a better predictor of carcass and meat quality traits compared with temperament assessment at the end of the feeding period [101].

## 7. Temperament vs. Some Immunological and Metabolic Blood Parameters Changes

Temperament also has an influence on immunological and metabolic indicators [49,102,103]. It remains related to the metabolic reaction after an experimental endotoxin injection [104]. Excitable calves had significantly higher serum CC, and after 85 days of feeding, they had noticeably lower daily growth rates than calm calves. Groups of calves of different temperaments were not different in terms of the total number of leukocytes as well as the result of differential blood count, lymphocyte proliferation, interferon production, the activity of NK cells, myeloperoxidase activity in neutrophils, lymphocyte subsets (CD4, CD8, or WC1) and IgA concentrations in the serum, whereas the IgM concentration was lower among calmer calves [103]. Correlations were discovered between cortisol and some hematological indicators, the average daily growth and the temperament of calves [104]. Moreover, in mature cattle with excitable temperament, there was a negative correlation with the Body Condition Score (BCS) [62,93,105]. The concentration of serum insulin in the calm group was higher than that in the nervous group [48]. For blood parameters, nervous cattle had greater values of cortisol and tended to have reduced serum insulin concentration compared to animals with calm temperament [97]. These cattle also had a higher level of acute phase proteins: haptoglobin and ceruloplasmin [105]. Some data suggest that nervous cattle may display limited behavioral signs of illness, which may prevent proper medical intervention, and increase the risk of transferring pathogens to healthy animals [106]. 

Investigation measuring cellular innate immune responses among calm and nervous Brahman bulls in response to handling and transportation were compared by Hulbert et. al. [73]. Transportation did not affect the leukocyte counts regardless of temperament, but neutrophil to mononuclear cell ratios were greater in nervous bulls compared to calm ones at 24 h after transportation. Similarly, serum glucose and CC were greater in nervous ones at 48 h after transportation. However, calm bulls tended to have elevated phagocytic and oxidative burst activity compared with nervous bulls at 48 h, which makes them less likely to be infected by different pathogens [73].

Blood serum from nervous steers contained a greater concentration of non-esterified fatty acids (NEFA), such as α-linoleic, γ-linoleic and eicosapentaenoic acids, compared with calm steers [107,108]. Excitable bulls may preferentially use alternative energy sources (i.e., NEFA) to a greater extent than those with calm and intermediate temperaments. In calm bulls, increases in blood urea nitrogen (BUN), glucose and insulin levels have been reported after lipopolysaccharide (LPS) administration, showing that bull temperament is associated with a metabolic response following an LPS-induced immune response [104]. Nervous bulls may allow for a quicker response to increasing energy demands required during times of immunological challenge compared with the time required to break down glycogen into glucose, which may enhance the immune response to LPS challenge. The use of circulating NEFA from lipolysis may reduce the negative metabolic consequences of an immune response by allowing a prompt response that increases energy demands required during an immunological challenge compared with the time required for glycogenolysis and gluconeogenesis. Consequently, nervous bulls can better handle moments of immune instability [108].

## 8. Temperament and Cattle Reproduction

### 8.1. Cows

Most studies examined the effects of temperament on reproduction in beef cattle [109]. Nellore cows with excitable temperament had a higher serum CC [110,111]. Moreover, more reactive cows had lower serum luteinizing hormone (LH) concentration [109], possibly due to the reduction of gonadotropin-releasing hormone (GnRH) pulses [67,112,113]. This may lead to a prolonged post-delivery anestrus phase [114,115]. Although impregnation can occur among excitable cattle as a result of decreased progesterone (P4) secretion and synthesis and increased release of prostaglandin F2α, early embryonic death may occur. Furthermore, timid cows may give birth to calves whose weight at birth will be lower, which suggests that the trait may have broader economic consequences than previously thought [115,116,117]. 

The calving interval of excitable beef cows was 24 days longer than the calm ones. Their pregnancy loss rate was also higher [78]. Similarly, the probability of getting pregnant at the beginning of the reproductive season and pregnancy near its end was higher among beef cows characterized by calm temperament than among the excitable ones [34,69,118]. Calm Nellore heifers showed a larger diameter of the dominant follicle on the day of timed artificial insemination (TAI) [110]. In addition, excitability may change the dynamics of ovarian follicles and increase estrus symptoms, which have no connection with the calving outcomes [14]. In excitable cows, there was a tendency to have smaller dominant follicles, smaller size of the corpus luteum (CL) and its diameter, and lower P4 concentrations on days 7 and 14 of the estrus cycle [105,111,119]. Guerson et al. [119] found that the excitable temperament in Nellore cows had an important endocrine effect reflected on the ovarian structures, with less follicular blood flow and a reduction in the volume of the preovulatory follicle and CL, however, without compromising the ovulation rate. Conversely, some studies indicate that the use of GnRH or long-acting P4, seven days after insemination, improves pregnancy rates in excitable animals and is a viable alternative to minimize the negative impact of stress and improve reproductive efficiency in beef cows [111]. 

The influence of cow excitability on the pregnancy rate after the embryo transfer (ET) procedure has not been widely researched [120,121]. Recently, some research has pointed to a significant influence of the temperament of recipients on the pregnancy rate after ET. It indicated that recipient heifers with calm temperament had a higher pregnancy rate (7.7% of points) in comparison to recipients with excitable temperament. Excitable cows that did not receive Flunixin Meglumine (FM) had a lower calving rate (at least 10.5%) in comparison to calm and excitable cows which received it [121]. In the assessment of temperament, only a two-stage scale and a subjective assessment of the EV of the recipients were used. Higher CC, P-substances (neuropeptide, which worked as a neurotransmitter and neuromodulator) and prostaglandin metabolites were noted in blood during ET and seven days later among excitable cows that had not received FM [23]. Those events caused embryo loses among some excitable cows, especially those that had not received FM. It seems that FM alleviates the effects of stress and maintains the P4 concentration, in consequence maintaining the pregnancy. A similar effect was achieved by administering aspirin and ibuprofen (both are strong COX-1 and/or COX-2 inhibitors) to recipient cows [120,121]. Many researchers [122,123,124] report that administering FM resulted in an increase in the pregnancy rate after transferring quality two embryos, whereas the effects were not achieved after transferring quality one embryos [123]. 

### 8.2. Bulls

In the calm Nellore bull, cortisol concentration was reduced compared to bulls with the reactive temperament. The serum testosterone concentration did not differ between groups with distinct behavior, even with increased serum CC and scrotal temperature. Moreover, semen quality (volume, kinetics, morphology and concentration) did not differ between the calm and reactive bulls [125]. Sperms of docile Angus bulls had a lower percentage of primary defects but more secondary defects than excitable bulls [126]. 

Many factors play a role in determining the libido of the bulls; these are genetics, environmental and individual factors. Some data indicate that temperament is likely to affect the expression of libido when animals are put into new situations [127]. Conversely, no difference in the libido score was observed in Sahiwal and Jersey × Sahiwal crossbred bulls [128]. The scrotal thermography in reactive bulls presented greater temperature at the caudal pole of the testicle. In Holstein–Fresian and Simmental bulls, only weak correlations were found between the behavioral traits of the bulls and the quality of their semen [129]. 

## 9. Conclusions

Temperament is one of the basic characteristics of any animal. In cattle, it has important economic implications. Cattle with calm temperament are more desirable than individuals whose temperament is described as aggressive or nervous. The latter are characterized by poorer health, lower milk production, lower weight gain, poorer meat quality and more problems with reproduction. They are also exposed to a higher risk of injury and pose a threat to other animals and humans. As a consequence, they have a higher probability of early culling. 

A number of scales have been developed to assess the temperament of cattle. Their main advantage is the fact that they can be performed relatively quickly in field conditions. They enable a fairly precise division of the assessed animals into categories ranging from calm individuals to nervous ones. At the same time, they guarantee satisfactory comparability. However, the developed tests are not an ideal solution and are not always repeatable in any conditions. Moreover, they are not widely known among breeders and farmers, and perhaps for this reason they are rarely used in practice. As a rule, breeders trust their own experience and critical observation of animals, eliminating individuals with undesirable temperaments, rather than professional tools designed to assess temperament. Perhaps for this reason, temperament scales are more useful in scientific research. Current research shows that there are some genes related to temperamental characteristics. Establishing their presence in cattle could be helpful in matching parental pairs. It would make it easier to eliminate individuals with nervous temperament earlier, thus, reducing the risk of undesirable traits in the offspring.

## Figures and Tables

**Table 1:** Selected temperament assessment methods in cattle.

Assessment Methods	Point Range	Evaluation Criteria	Beef/Dairy Cattle	Reference
Modified two-point chute exit and gait score	0 = calm, slow exit and walk 1 = excitable, fast exit or jump or trot or run	Chute exit	Beef	Kasimanickam et al., 2014 [34]
Chute exit	High responders—less than 2 s Medium responders—2–4 s Low responders—more than 4 s	Chute exit	Dairy	Sutherland et al., 2012 [24]
	1 = calm animals 2 = normal 3 = excitable or aggressive	Behavior rated during milking	Dairy	Kalińska, Slósarz 2016 [26]
	1 = mild, an animal which stands very quietly on the scale 2 = slightly restless, average temperament, an animal which stands quietly but moves frequently 3 = restless, an animal which moves almost continuously and is difficult to weigh 4 = nervous, a restless animal which struggles violently and is very difficult to weigh	Behavior rated in the weighing scale	Beef	Sato S. 1981 [28]
Modification of Tulloh (1961)	1 = docile 2 = slightly restless 3 = restless 4 = nervous 5 = wild	Behavior rated in the weighing scale	Beef	Kabuga et al., 1992 [29]
	1 = calm 2 = slightly restless 3 = squirming 4 = continuous, very vigorous movement and shaking of squeeze chute 5 = rearing, twisting of the body and struggling violently	Behavior rated in squeeze chute	Beef/Dairy	Grandin 1993 [25]
	1–5 (docile to nervous) 1—docile, very quiet, never gives any trouble, extremely docile during milking and preparation, the “ideal milker” 2—slightly restless, stands quietly, not bothered by preparation or milking 3—restless, generally quiet but moves around a lot 4—aggressive, appears very restless during preparation or milking 5—nervous, appears very restless during preparation or milking, struggles violently	Behavior rated during milking	Dairy	Choudhary et al., 2017 [27]
	1–5 (calm-excited)	Pen score—behavior as the handler approached group	Beef	King et al., 2006 [30]
	1 (docile)–6 (aggressive)	30 s of response to a human stressor	Beef	Parham et al., 2022 [31]

## Data Availability

Not applicable.

## References

[1] Kaleta T. (2014). Osobowość zwierząt: Krótki przegląd współczesnych badań. Życie Weter..

[2] Waśniewski H. (1893). Charakter Zwierząt.

[3] Scott J.P., Fredericson E. (1951). The causes of fighting in mice and rats. Physiol. Zool..

[4] Stricklin W.R., Kautz-Scanavy C.C. (1984). The role of behavior in cattle production: A review of research. Appl. Anim. Ethol..

[5] Chang Y., Brito L.F., Alavarenga A., Wang Y.B. (2020). Incorporating temperament traits in dairy cattle breeding programs: Challenges and opportunities in the phenomics era. Anim. Front..

[6] Marçal-Pedroza M.G., Campos M.M., Pereira L.G.R., Machado F.S., Tumich T.R., Paranhos da Costa M.J.R., Sant’Anna A.C. (2020). Consistency of temperamental traits and their relationship with milk yeld in lactcting prmiparous F1 Holstein-Gyr cows. Appl. Anim. Behav. Sci..

[7] Burrow H.M., Dillon R.D. (1997). Relationships between temperament and growth in a feedlot and commercial carcass traits of Bos Indicus crossbreds. Aust. J. Exp. Agric..

[8] Burrow H.M., Seifert G.W., Corbet N.J. (2000). Genetic and environmental factors affecting temperament of zebu and zebu-derived beef cattle grazed at pasture in the tropics. Aust. J. Agric. Res..

[9] Sebastian T., Watts J.M., Stookey J.M., Buchanan F., Waldner C. (2011). Temperament in beef cattle: Methods of measurement and their relationship to production. Can. J. Anim. Sci..

[10] Waiblinger S., Boivin X., Pedersen V., Tosi M.-V., Janczak A.M., Viser E.K., Jones R.B. (2006). Assessing the human–animal relationship in farmed species: A critical review. Appl. Anim. Behav. Sci..

[11] Ordloff D. (2001). Introduction of electronics into milking technology. Comput. Electron. Agric..

[12] Wethal K.B., Heringstad B. (2019). Genetic analyses of novel temperament and milk ability traits in Norwegian Red cattle based on data from automatic milking system. J. Dairy Sci..

[13] Szymik B., Topolski P., Jagusiak W. (2021). Genetic parameters of workability traits in the population of polish Holstein-Fresian Cows based on conventional and genomic data. Animals.

[14] Cooke R.F., Schubach K.M., Marques R.S., Peres R.F.G., Silva L.G.T., Carvallho R.S., Cipriano R.S., Bohnert D.W., Pires A.V., Vascontelos J.L.M. (2017). Effects of temperament on physiological, productive, and reproductive responses in beef cows. J. Anim. Sci..

[15] Lee C., Café L.M., Robinson S.L., Doyle R.E., Lea J.M., Small A.H., Colditz I.G., Allison M. (2018). Anxiety influences attention bias but not flight speed and crush score in beef cattle. Appl. Anim. Behav. Sci..

[16] Mackay J., Haskell M.J. (2015). Consistent Individual Behavioural Variation: The Difference between Temperament, Personality and Behavioural Syndromes. Animals.

[17] Neave H.W., Costa J.H.C., Weary D.M., Keyserlink M.A.G. (2020). Long-term consistency of personality traits of cattle. R. Soc. Open Sci..

[18] Shahin M. (2018). The effects of positive human contact by tactile stimulation on dairy cows with different personalities. Appl. Anim. Behav. Sci..

[19] Hedlund L., Løvlie H. (2015). Personality and production: Nervous cows produce less milk. J. Dairy Sci..

[20] Foris B., Zebunke M., Langbaum J., Melzer N. (2012). Evaluation the temporal and situational concidtency of personality traits in adult dairy cattle. PLoS ONE.

[21] Buss A.H. (1995). Personality: Temperament, Social Behavior and the Self.

[22] Finkemeier M.A., Langbein J., Puppe B. (2018). Personality Research in Mammalian Farm Animals: Concepts, Measures, and Relationship to Welfare. Front. Vet. Sci..

[23] Kasimanickam R.K., Hall J.B., Estill C.T., Kastelic J.P., Joseph C., Abdel Aziz R.L., Nak D. (2017). Flunixin meglumine improves pregnancy rate in embryo recipient beef cows with an excitable temperament. Theriogenology.

[24] Sutherland M.A., Rogers A.R., Verkerk G.A. (2012). The effect of temperament and responsiveness towards humans on the behavior, physiology and milk production of multi-parous dairy cows in a familiar and novel milking environment. Physiol. Behav..

[25] Grandin T. (1993). Behavioral agitation during handling of cattle is persistent over time. Appl. Anim. Behav. Sci..

[26] Kalińska A., Slósarz J. (2016). Influence of cow temperament and milking speed on herd life, lifetime milk yield and reasons of cow culling Annals of Warsaw University of Life Sciences–SGGW. Anim. Sci..

[27] Choudhary K.K., Bharadway A., Jerome A., Khanna S. (2017). Relationship of temperament with oestrous behaviour, resumption of ovarian cyclicity and milk yield in post-partum Murrah buffaloes. Reprod. Domest. Anim..

[28] Sato S. (1981). Factors associated with temperament of beef cattle. Jpn. J. Zoot. Sci..

[29] Kabuga J.D., Appiah P. (1992). A note on the ease of handling and flight distance of Bos indicus, Bos taurus and their crossbreds. Anim. Prod..

[30] King D.A., Schuehle Pfeiffer C.E., Randel R.D., Welsh T.H., Oliphint R.A., Baird B.E., Curley K.O., Vann R.C., Hate D.S., Savell J.W. (2006). Influence of animal temperament and stress responsiveness on the carcass quality and beef tenderness of feedlot cattle. Meat Sci..

[31] Parham J.T., Tanner A.E., Blevins S.R., Wahlberg M.L., Lewis R.M. (2022). Cattle acclimate more substantially to repeated handling when confined individually in a pen than when assessed as a group. J. Anim. Sci..

[32] Fordyce G., Goddard M.E. (1984). Maternal influence on the temperament of Bos indicus cows. Anim. Prod. Aust..

[33] Fordyce G., Dodt R.M., Wythes J.R. (1988). Cattle temperaments in extensive beef herds in northern Queensland. 1. Factors affecting temperament. Aust. J. Agric. Res..

[34] Kasimanickam R., Schroeder S., Assay M., Kasimanickam V., Moore D.A., Gay J.M., Whittier W.D. (2014). Influence of temperament score and handling facility on stress, reproductive hormone concentrations, and fixed time AI pregnancy rates in beef heifers. Reprod. Domest. Anim..

[35] Purcell D., Arave C.W., Walters J.L. (1988). Relationship of three measures of behaviour to milk production. Appl. Anim. Behav. Sci..

[36] Whay H.R., Main D.C.J., Green L.E., Webster A.J.F. (2003). Assessment of the welfare of dairy cattle using animal-based measurements: Direct observations and investigation of farm records. Vet. Rec..

[37] Burrow H.M., Siefert G.W., Corbet N.J. (1988). A new technique for measuring of temperament in cattle. Proc. Aust..

[38] Curley Jr K.O., Paschal J.C., Welsh Jr T.H., Randel R.D. (2006). Technical note: Exit velocity as a measure of cattle temperament is repeatable and associated with serum concentration of cortisol in Brahman bulls. J. Anim. Sci..

[39] Piovezan U., Cyrillo J.N.S.G., Costa M.J.R.P. (2013). Breed and selection line differences in the temperament of beef cattle. Acta Sci. Anim. Sci..

[40] Agravat P.H., Patbandha T.K., Odera M.D., Savsani H.H., Ahlawat A.R., Sabapara G.P., Maurya P., Kalasava S.K., Belim S.Y., Sanchaniya J.M. (2022). Association of temperament with temperament index, exit score and parlour leaving speed in Gir Cows. J. Pharm. Innov..

[41] Sant’Anna A.C., Paranhos da Costa M.J.R. (2013). Validity and feasibility of qualitative behavior assessment for the evaluation of Nellore cattle temperament. Livest. Sci..

[42] Smolinger J., Škorjanc D. (2021). Methods of Assessing Cattle Temperament and Factors Affecting it: A review. Agricultura.

[43] Kovács L., Tőzsér J., Szenci O., Póti P., Kézér F.L., Ruff F., Gábriel-Tőzsér G., Hoffmann D., Bakony M., Jurkovich V. (2014). Cardiac responses to palpation per rectum in lactating and nonlactating dairy cows. J. Dairy. Sci..

[44] Brandao Poggi A., Cooke R.F. (2021). Effects of Temperament on the Reproduction of Beef Cattle. Animals.

[45] Kovács L., Tőzsér J., Kézér F.L., Ruff F., Aubin-Wodala M., Albert E., Choukeir A., Szelényi Z., Szenci O. (2015). Heart rate and heart rate variability in multiparous dairy cows with unassisted calvings in the periparturient period. Physiol. Behav..

[46] Kovács L., Kézér F.L., Tőzsér J., Szenci O., Póti P., Pajor F. (2015). Heart Rate and Heart Rate Variability in Dairy Cows with Different Temperament and Behavioural Reactivity to Humans. PLoS ONE.

[47] Frondelius L., Järvenranta K., Koponen T., Mononen J. (2015). The effects of body posture and temperament on heart rate variability in dairy cows. Physiol. Behav..

[48] Keykhasaber M., Yosofelhani M., Mirzaei H., Rokouei M., Dahgani M. (2017). Relationship of temperament with performance, body shape indices and some blood parameters in Sistani calves. Anim. Sci. J..

[49] Erdmann S., Mohr E., Derno M., Tuchscherer A., Schäff C., Börner S., Kautzsch U., Kuhla B., Hammon H.M., Röntgen M. (2018). Indices of heart rate variability as potential early markers of metabolic stress and compromised regulatory capacity in dried-off high-yielding dairy cows. Animal.

[50] Marino L., Allen K. (2017). The Psychology of Cows. Anim. Behav. Cogn..

[51] Gómez Y., Bieler R., Hankele A.K., Zähner M., Savary P., Hillmenn E. (2018). Evaluation of visible eye white and maximum eye temperature as non-invasive indicators of stress in dairy cows. Appl. Anim. Behav. Sci..

[52] Sandem A., Braastad B., Boe K. (2002). Eye white may indicate emotional state on a frustration-contentedness axis in dairy cows. Appl. Anim. Behav. Sci..

[53] Core S., Widowski T., Mason G., Miller S. (2009). Eye white percentage as a predictor of temperament in beef cattle. J. Anim. Sci..

[54] Lanier J., Grandin T., Green R., Avery D., McGee K. (2000). The relationship between reaction to sudden, intermittent movements and sounds and temperament. J. Anim. Sci..

[55] Burrow H.M. (1997). Measurement of temperament and their relationship with performance traits of beef cattle. Anim. Breed. Abstr..

[56] Grandin T. (1994). Solving livestock handling problems. Vet. Med..

[57] Shen J.F., Chen Q.M., Zhang F.W., Hanif Q., Huang B.Z., Chen N.B., Qu K.X., Zhan J.X., Chen H., Jiang Y. (2022). Genome-wide association study identifies quantitative trait loci affecting cattle temperament. Zool. Res..

[58] Jakimowicz M., Szyda J., Zarnecki A., Jagusiak W., Morek-Kopeć M., Kosińska-Selbi B., Scuchocki T. (2022). Genome-Wide Genomic and Functional Association Study for Workability and Calving Traits in Holstein Cattle. Animals.

[59] Paredes-Sanchez F.A., Sifuentes-Rincon A.M., Casas E., Arelano-Vera W., Para-Bracamonte M., Riley D.G., Welsh T.H., Randel R.D. (2020). Novel genes involvee in the genetic architecture of tepmerqament in Brachman Cattle. PLoS ONE.

[60] Hoppe S., Brandt H.R., Konig S., Erhardt G., Gauly M. (2010). Temperament traits of beef calves measured under field conditions and their relationships to performance. J. Anim. Sci..

[61] Voisinet B.D., Grandin T., Tatum J.D., O’Connor S.F., Struthers J.J. (1997). Feedlot cattle with calm temperaments have higher average daily gains than cattle with excitable temperaments. J. Anim. Sci..

[62] Voisinet B.D., Grandin T., O’Connor S.F., Tatum J.D., Deesing M.J. (1997). Bos indicus-cross feedlot cattle with excitable temperaments and tougher meat and higher incidence of borderline dark cutters. Meat Sci..

[63] Tözsér J., Maros K., Szentleléki A., Zándoki R., Nikodémusz E., Balázs F., Bailo A., Alföldi L. (2003). Evaluation of temperament in cows of different age and bulls of different colour variety. Czech J. Anim. Sci..

[64] Keller C.E. (1947). Coat colour, physique and temperament. J. Hered..

[65] Prasad M.R.V., Jaya Laxmi P., Screenivas Kumar D. (2011). Temperament of Murrah buffaloes in different lactations and its effect on milk yield. Indian J. Anim. Res..

[66] Karamfilov S. (2022). Study on the temperament of cows of the Aberdeen Angus cattle breed. Czech J. Anim. Sci..

[67] Cooke R.F., Arthington J.D., Austin B.R., Yelich J.V. (2009). Effects of acclimation to handling on performance, reproductive, and physiological responses, of Brahman-crossbred heifers. J. Anim. Sci..

[68] Cooke R.F., Bohnert D.W., Meneghetti M., Losi T.C., Vasconcelos J.L.M. (2011). Effects of temperament of pregnancy rates to fixed-time AI in Bos indicus beef cows. Livest. Sci..

[69] Cooke R.F., Bohnert D.W., Cappellozza B.I., Mueller C.J., Del Curto T. (2012). Effects of temperament and acclimation to handling on reproductive performance of Bos taurus beef cattle. J. Anim. Sci..

[70] Hearnshaw H., Morris C.A. (1984). Genetic and environmental effects of a temperament score in beef cattle. Aust. J. Agric. Res..

[71] Hanna L.L.H., Hieber J.K., Yu H., Celestino E.F., Dahlen C.R., Wagner S.A., Riley D.G. (2019). Blood collection has negligible impact on scoring temperament in Angus-based weaned calves. Livest. Sci..

[72] Hulbert L.E., Carroll J.A., Burdick N.C., Randel L.D., Brown M.S., Ballou M.A. (2011). Innate immune responses of temperamental and calm cattle after transportation. Vet. Immunol. Immunopathol..

[73] Hedlund L. (2013). Personality and Production in Dairy Cows. Master’s Thesis.

[74] Gart E.V., Lawton S.D., Suchodolski J.S., Randel R.D., Welsh T.H. (2019). The relationship of weaning stress, sex and temperament on fecal microbiota and metabolites in Brahman calves. J. Anim. Sci..

[75] Mir R.A., Kleinhenz M.D., Coetzee J.F., Allen H.K., Kudva I.T. (2019). Fecal microbiota changes associated with dehorning and castration stress primarily affects light-weight dairy calves. PLoS ONE.

[76] Bruno K., Vanzat E., Vanzat K., Altman A., Kudupoje M., McLeod K. (2018). Relationship between quantitative measures of temperament and other observed behaviors in growing cattle. Appl. Anim. Behav. Sci..

[77] Chen X., Ogdahl W., Hulsman L.L., Dahlen H.C.R., Riley D.G., Sarah G.R., Wagner A., Berg E.P., Sun X. (2021). Evaluation of beef cattle temperament by eye temperature using infrared thermography technology. Comput. Electron. Agric..

[78] Kasimanickam V.R., Abdel Aziz R.L., Williams H.M., Kasimanickam R.K. (2018). Predictors of beef calf temperament at weaning and its impact on temperament at breeding and reproductive performance. Reprod. Domest. Anim..

[79] Reddy A.O., Tripathi V.N. (1987). Studies on temperament on Murrah buffaloes. Indian J. Anim. Prod. Mgmt..

[80] Mincu M., Gavojdian D., Nicolae I., Olteanu N.C., Vlagioiu C. (2021). Effects of milking temperament of dairy cows on production and reproduction efficiency under tied stall housing. J. Vet. Behav..

[81] Morales-Piñeyrua J.T., Damian J.P., Banchero G., Blache D., Sant’Anna A. (2022). Metabolic profile and productivity of dairy Holstein cows milked by a pasture-based automatic milking system during early lactation: Effects of cow temperament and parity. Res. Vet. Sci..

[82] Neave H.W., Zobel G., Thoday H., Saunders K., Edwards J.P., Webster J. (2022). Toward on-farm measurement of personality traits and their relationships to behavior and productivity of grazing dairy cattle. J. Dairy Sci..

[83] Sawa A., Bogucki M., Neja W., Krężel-Czopek S. (2017). Effect of temperament on performance of primiparous dairy cows. Ann. Anim. Sci..

[84] Shutherhand M.A., Dowlng S.K. (2014). The relationship between responsiveness of first lactation heifers to humans and behavioral response to milking and milk production measures. J. Vet. Bahav..

[85] Marçal-Pedroza M.G., Canozi M.E.A., Campos M.M., Sant’Anna A.C. (2023). Effects of dairy cow temperament on milk yield: A systematic review and meta-analysis. J. Anim. Sci..

[86] Van Reenen C.G., Van der Werf J.T.N., Bruckmaier R.M., Hopster H., Engel B., Noordhuizen J.P.T.M., Blokhuis H.J. (2002). Individual differences in behavioral and physiological responsiveness of primiparous dairy cows to machine milking. J. Dairy Sci..

[87] Dickson D.P., Barr G.R., Johnson L.P., Wieckert D.A. (1970). Social Dominance and Temperament of Holstein Cows. J. Dairy Sci..

[88] Orbán M., Gaál K.K., Pajor F., Szentléleki A., Póti P., Tőzsér J., Gulyás L. (2011). Effect of temperament of Jersey and Holstein Friesian cows on milk production traits and somatic cell count. Arch. Anim. Breed..

[89] Rupp R., Boichard D. (1999). Genetic parameters for clinical mastitis, somatic cell score, production, udder type traits, and milking ease in first lactation Holsteins. J. Dairy Sci..

[90] Van Doormaal B., Trends in Disposal Reasons Canadian Dairy Network. www.cdn.ca/articles.php.

[91] Szymik B., Topolski P., Jagusiak W. (2015). Phenotypic characteristics of workability traits in the population of Polish Black- and Red-and-White Holstein-Friesian, Simmental and Polish Red cows. Rocz. Nauk. Zootech..

[92] Llonch P., Somarriba M., Duthie C.A., Troy S., Roehe R., Rooke J., Haskell M.J., Turner S.P. (2018). Temperament and dominance relate to feeding behaviour and activity in beef cattle: Implications for performance and methane emissions. Animal.

[93] Café L.M., Robinson D.L., Ferguson D.M., McIntyre B.L., Geesink G.H., Greenwood P.L. (2011). Cattle temperament: Persistence of assessments and associations with productivity, efficiency, carcass and meat quality traits. J. Anim. Sci..

[94] Boles J.A., Kohlbeck K.S., Meyers M.C., Perz K.A., Davis K.C., Thomson J.M. (2015). The use of blood lactate concentration as an indicator of temperament and its impact on growth rate and tenderness of steaks from Simmental × Angus steers. Meat Sci..

[95] Bourguet C., Deiss V., Gobert M., Durand D., Boissy A., Terlouw E.M.C. (2010). Characterising the emotional reactivity of cows to understand and predict their stress reactions to the slaughter procedure. Appl. Anim. Behav. Sci..

[96] Nkrumah J.D., Crews Jr D.H., Basarab J.A., Price M.A., Okine E.K., Wang Z., Li C., Moore S.S. (2007). Genetic and phenotypic relationships of feeding behavior and temperament with performance, feed efficiency, ultrasound, and carcass merit of beef cattle. J. Anim. Sci..

[97] Francisco C.L., Resende F.D., Benatti J.M.B., Casthilhos A.M., Cooke R.F., Jorge A.M. (2015). Impacts of temperament on Nellore cattle: Physiological responses, feedlot performance, and carcass characteristics. J. Anim. Sci..

[98] Holmes J.H.K., Ashmore C.R., Robinson D.W. (1972). Effects of stress on cattle with hereditary muscular hypertrophy. J. Anim. Sci..

[99] De Moura S.V., Silveira I.D.B., Ferreira O.G.L., Mendoca F.S., Moreira S.M., Restle R., Garcia A.A.B., Vaz R.Z. (2021). Lairage periods on temperament score and meat quality of beef cattle. Anim. Sci..

[100] León-Llanos L.M., Flórez-Díaz H., Duque-Muñoz L.G., Villarroel M., Miranda-de la Lama G.C. (2022). Influence of temperament on performance and carcass quality of commercial Brahman steers in a Colombian tropical grazing system. Meat Sci..

[101] Sant’anna A.C., Da Silva Valente T., Braga Magalhaes A.F., Espigolan R., Ceballos M.C., Galvao De Albuquergue L., Paranhos da Costa M.J.R. (2019). Relationships between temperament, meat quality, and carcass traits in Nellore cattle. J. Anim. Sci..

[102] Burdick N.C., Randel R.D., Carroll J.A., Welsh T.H. (2011). Interactions between Temperament, Stress, and Immune Function in Cattle. Int. J. Zool..

[103] Fell L.R., Colditz I.G., Walker K.H., Watson D.L. (1999). Associations between temperament, performance and immune function in cattle entering a commercial feedlot. Aust. J. Exp. Agric..

[104] Burdick Sanchez N.C., Carroll J.A., Randel R.D., Vann R.C., Welsh T.H. (2014). Associations between endotoxin-induced metabolic changes and temperament in Brahman bulls. J. Anim. Physiol. Anim. Nutr..

[105] Vedowatto M., Faria F.J.C., Csta D.S., Cooke R.F., Sanchez J.M.D., Moriel P., Coelho L.M., Franco G.L. (2021). Effects of temperament on body parameters, ovarian structures and inflammatory response in grazing Nellore cows following fixed-time artificial insemination. J. Vet. Behav..

[106] Burdick Sanchez N.C., Carroll J.A., Welsh T.H., Randel R.D., Vann R.C. (2012). Metabolic Differences in Cattle with Excitable Temperaments Can Influence Productivity. Animal and Dairy Sciences Department Report.

[107] Gardner T., Legako J.F., Burdick Sanchez N.C., Broadway P.R., Carroll J.A., Vann N.C. (2016). Influence of cattle temperament on blood serum fatty acid content. J. Anim. Sci..

[108] Burdick Sanchez N.C., Carrol J.A., Broadway P.R., Hughes H.D., Roberts S.L., Richeson J.T., Schmidt T.B., Vann R.C. (2016). Cattle temperament influences metabolism: Metabolic response to glucose tolerance and insulin sensitivity tests in beef steers. Domest. Anim. Endocrinol..

[109] Echternkamp S.E. (1984). Relationship between LH and cortisol in acutely stressed beef cows. Theriogenology.

[110] Mello B.P., Filho M.M., Lemes K.M., Goncalvez R.L., Mendes Lollato P., Zanella A.J., de Vasconcelos Ferreira T.F., Puglies G., Hoffman Mandureira E., Gonella-Diaza A. (2020). Importance of temperament in the pregnancy by timed insemination in bovine females *Bos taurus indicus*. Livest. Sci..

[111] Couto S.R.B., Guerson Y.B., Caparelli M.M.P.M., Andrade J.P.N., Jacob J.C.F., Barbero R.P., Mello M.R.B. (2022). Mitigation of low pregnancy rate in excitable Nellore cows by administration of GnRH or P4. Theriogenology.

[112] Dobson H., Smith R.F. (2000). What is stress, and how does it affect reproduction?. Anim. Reprod. Sci..

[113] Cooke R.G., Benhaj K.M. (1989). Effects of ACTH and cortisol on luteolysis in the ewe. Anim. Reprod. Sci..

[114] Dobson H.A., Ribadu A.Y., Noble K.M., Tebble J.E., Ward W.R. (2000). Ultrasonography and hormone profiles of adrenocorticotrophic hormone (ACTH)-induced persistent ovarian follicles (cysts) in cattle. J. Reprod. Fertil..

[115] Turner S.P., Jack M.C., Lawrence A.B. (2013). Precalving temperament and maternal defensiveness are independent traits but precalving fear may impact calf growth. J. Anim. Sci..

[116] Berry E.A., Scrivens M., Hillerton J.E. (2005). Milking machine test survey of UK herds. Vet. Rec..

[117] Sewalem A., Miglior F., Kistemaker G.J. (2010). Analysis of the relationship between workability traits and functional longevity in Canadian dairy breeds. J. Dairy Sci..

[118] Kasimanickam R., Asay M., Schroeder S., Kasimanickam V., Gay J.M., Kastelic J.P., Hall J.B., Whittier W.D. (2014). Calm temperament improves reproductive performance of beef cows. Reprod. Domest. Anim..

[119] Guerson Y.B., Couto S.R.B., Cassia R.D.C.L., Grillo G.F., Jacob J.F.C., Barbato R.P., Mello M.R.B. (2021). Vascular perfusion and the volume of the preovulatory follicle are affected by the temperament of Nellore cows. Livest. Sci..

[120] Cheng Z., Nolan A.M., McKellar Q.A. (1998). Measurement of cyclooxygenase inhibition in vivo: A study of two non-steroidal anti-inflammatory drugs in sheep. Inflammation.

[121] Jaśkowski B.M., Opałka A., Herudzińska M., Czeladko J., Baumgartner W., Jaśkowski J.M. (2021). A critical overview on prostaglandin inhibitors and their influence on pregnancy results after insemination and embryo transfer in cows. Animals.

[122] Scenna F.N., Hockett M.E., Towns T.M., Saxton A.M., Rohrbach N.R., Wehrman M.E., Schrick F.N. (2005). Influence of a prostaglandin synthesis inhibitor administered at embryotransfer on pregnancy rates of recipient cows. Prostagl. Other Lipid Mediat..

[123] Pugh M.L., Moreira M.B., Gilbert G.R., Youngs C.R. Influence of prostaglandin F2 synthesis inhibitors on pregnancy rate of embryo transfer recipient heifers. Proceedings of the 15th International Congress on Animal Reproduction.

[124] Elli M., Gaffuri B., Frigerio A., Zanardelli M., Covini D., Candiani M., Vignali M. (2001). Effect of a single dose of ibuprofen lysinate before embryo transfer on pregnancy rates in cows. Reproduction.

[125] Braz K.M.G., Morato Monteneiro F., Gomes Fernandes L., Nantes Rodriguez N., da Cuha Peixoto K., Green L.E., Cortez A., Crespilho A.M. (2020). Does bull temperament impact growth performance and semen quality?. Livest. Sci..

[126] Lockwood S.A., Kattesh H.G., Rhinehart J.D., Strickland L.G., Krawczel P.D., Wilkerson J.B., Krikpatrick F.D., Saxton A.M. (2017). Relationships among temperament, acute and chronic cortisol and testosterone concentrations, and breeding soundness during performance testing of Angus bulls. Theriogenology.

[127] Petherick J.C. (2005). A review of some factors affecting the expression of libido in beef cattle, and individual bull and herd fertility. Appl. Anim. Behav. Sci..

[128] Vijaya Bhaskara Reddy M., Sasikala P. (2013). Sexual behaviour of Sahiwal and Jersey X Sahiwal bulls in tropical environments at villages of Chittor district. Int. J. Adv. Sci. Technol. Res..

[129] Adamczyk K., Makowska A., Jedraszczyk J., Hebda L., Gil Z. (2013). Effect of behaviour of Holstein-Friesian and Simmental bulls on semen quality. J. Cent. Eur. Agric..

